# Synergistic role of *GhGCS1* in cotton root development and verticillium wilt resistance

**DOI:** 10.1007/s44154-026-00293-6

**Published:** 2026-02-06

**Authors:** Qiaoling Wang, Xingying Yan, Li Huang, Qi Niu, Ming Luo, Fan Xu

**Affiliations:** 1 College of Agronomy and Biotechnology, Southwest University, Chongqing, China; 2 Engineering Research Center of South Upland Agriculture, Ministry of Education, Southwest University, Chongqing, China

**Keywords:** Cotton, Sphingolipid, Root development, Verticillium wilt

## Abstract

Sphingolipids are not only essential structural components of cellular membranes but also key signaling molecules that regulate plant growth, development, and stress responses. However, their specific roles in root development remain largely unknown. In the presented study, we demonstrate that overexpression of the *GhGCS1* gene significantly enhances lateral root development in cotton. Integrated comprehensive transcriptome analysis and phytohormone quantification revealed that *GhGCS1* promotes lateral root initiation and elongation primarily by suppressing cytokinin biosynthesis. Notably, *GhGCS1* overexpression also markedly improved cotton resistance to *Verticillium dahliae*. Further molecular analyses indicated that *GhGCS1* enhances cotton verticillium wilt resistance through modulation of the expression of sphingolipid-associated brassinosteroid- and pathogenesis-related genes. Collectively, these findings reveal a dual regulatory role for *GhGCS1* in coordinating root architecture and immune responses in cotton, providing novel insights and potential strategies for developing crop varieties with improved yield potential and stress tolerance.

**Supplementary Information:**

The online version contains supplementary material available at 10.1007/s44154-026-00293-6.

## Introduction

Cotton (*Gossypium* spp.) is one of the most important natural fiber crops worldwide and has played an indispensable role in the development of human civilization. Cotton fibers account for more than 60% of global textile raw materials and are essential for the production of clothing, home furnishings, and industrial fabrics. Beyond fiber productions, cotton seeds are processed into vegetable oil and high-protein animal feed, while cotton stalks are utilized for biomass energy generation, enabling comprehensive utilization of cotton resources. Cotton production and its downstream industries support the livelihoods of approximately 250 million people and constitute a major agricultural sector in leading cotton-producing countries, including China, the United States, and India (Huang et al. 2021; Wen et al. 2023).

Roots are essential plant organs responsible for the uptake of water and mineral nutrients and play a fundamental role in overall plant growth and development (Karlova et al. 2021). A typical taproot system comprises a primary root, lateral roots, and adventitious roots. The primary root typically originates from the embryonic radicle of the seeds (Petricka et al. 2012). Subsequently, lateral roots emerge in a rhythmic, sequential manner along the primary root, often synchronized with the circadian cycle (Moreno-Risueno et al. 2010). Most roots develop below-ground, forming the subterranean portion of the plant that provides anchorage and structural support for the above-ground portion (Petricka et al. 2012). In addition to their developmental roles, roots are critical for enabling plants to withstand a wide range of environmental stresses (Karlova et al. 2021). However, the molecular mechanisms governing cotton root system development and their functional roles in mediating the response of cotton to environmental stresses remain incompletely understood.

The plasma membrane (PM), which surrounds each cell, functions as a dynamic interface that facilitates communication between individual cells and their environment, as well as across the entire organism (Breslow and Weissman 2010). Sphingolipids and sterols are highly enriched in specialized PM microdomains, commonly known as lipid rafts, where they primarily contribute to membrane organization and structural integrity (Cacas et al. 2016). Sphingolipids are composed of three core structural elements: a long-chain base (LCB), typically derived from sphingosine; a long-chain or a very long-chain fatty acid (VLCFA); and a polar head group (Yang et al. 2025b). Beyond their structural roles, sphingolipids have emerged as important signaling platforms that participate in diverse cellular processes, including plant development, stimulus perception, and stress responses (Liu et al. 2021; Haslam et al. 2022).

A chemical genetic screen revealed that iminosugar inhibitors of plant glucosylceramide synthase suppress root growth in Arabidopsis and cereal species (Rugen et al. 2018). Partial RNA interference (RNAi)-mediated suppression of *AtLCB1* expression is associated with a marked reduction in plant size and altered leaf morphology, primarily due to impaired cell expansion caused by elevated levels of saturated sphingolipid LCBs (Chen et al. 2006). Similarly, T-DNA double mutants and RNAi-suppressed lines of the two *Arabidopsis thaliana* LCB C-4 hydroxylase genes, *Sphingoid Base Hydroxylase 1* (*SBH1*) and *SBH2*, exhibit growth reductions that correlate with the reduced levels of trihydroxy LCBs in sphingolipids (Chen et al. 2008). In rice, single-gene mutations in *OsIPCSs* impair growth and development, whereas mutations in multiple *OsIPCS* genes result in more severe dwarf phenotypes (Wang et al. 2023). In soybean, overexpression of *GmSLD1*, which encodes an LCB Δ8 desaturase, induces strong seed dormancy by reducing the gibberellin to abscisic acid ratio in seeds (Gao et al. 2025). Sphingolipidomic analysis of cotton fibers have revealed that phytoceramide species containing hydroxylated and saturated VLCFAs play critical roles in fiber elongation (Chen et al. 2021). Consistently, LCB hydroxylation affects plant growth and callose deposition in *Physcomitrium patens* (Gömann et al. 2021). In cotton, overexpression of the ceramide synthase gene *GhCS1* inhibits fiber cell initiation and elongation by promoting the synthesis of ceramides containing dihydroxy LCBs and VLCFAs (Li et al. 2022). In contrast, down-regulation of the fiber-specific KCR-like gene *GhKCRL1* suppresses fiber elongation by blocking sphingolipid biosynthesis in fiber cells (Meng et al. 2022). The cotton sphingosine kinase *GhLCBK1* is involved in fiber cell elongation through its regulatory effects on sphingosine-1-phosphate and auxin biosynthesis (Zhang et al. 2024). However, the roles of sphingolipids in plant root development remain poorly understood and require further investigation.

Sphingolipids play critical roles in plant responses to both abiotic and biotic stresses. A higher plant Δ8 sphingolipid desaturase enhances tolerance to aluminum toxicity and low-temperature stress in yeast and plants (Ryan et al. 2007; Chen et al. 2011). The *sld1 sld2* double mutants exhibit enhanced resistance to the fungal wilt pathogen *Verticillium dahliae* (*V. dahliae*) and the bacterial pathogen *Pseudomonas syringae* pv. tomato DC3000, a phenotype associated with increased plasmodesmata (PD)-associated callose deposition and reduced PD permeability (Liu et al. 2020). In rice, overexpression of the glycosylinositol phosphorylceramide (GIPC) glycosyltransferase gene *OsGMT1* suppresses plant immune responses and delays heading time (Lin et al. 2023). GCD1 and GCD3 catalyze the hydrolysis of glucosylceramides (GlcCers) into ceramides, resulting in the removal of specific GlcCers from the PM and their subsequent replacement with GIPCs. This remodeling alter membrane biophysical properties, thereby improving plant acclimation to osmotic stress and influencing resistance to *Spodoptera exigua* (Li et al. 2025b). The balance between ceramide and ceramide-1-phosphate (C1P) is also critical for plant defense against *Sclerotinia sclerotiorum*. CERK mutant lines display increased susceptibility, whereas CERK overexpression lines exhibit enhanced resistance (Ouyang et al. 2025). We previously demonstrated that fumonisin B1 (FB1), a sphingolipid biosynthesis inhibitor, is produced by *V. dahliae* and likely functions as a virulence factor contributing to verticillium wilt symptom development in cotton (Xu et al. 2022), suggesting an important role for sphingolipids in this disease. Notably, we also found that overexpression of the sphingolipid-related gene *GhIQD10* enhances cotton resistance to verticillium wilt, albeit at the cost of reduced fiber length (Xu et al. 2022, 2023). Collectively, these findings highlight a trade-off between sphingolipid-mediated developmental regulation and disease resistance, underscoring the need for further investigation.

We previously demonstrated that GhGCS1 functions as a glycosylceramide (GluCer) synthase. Using transgenic validation and exogenous application assays, we further confirmed that *GhGCS1* and its product GluCer participated in regulating cotton fiber elongation by modulating brassinosteroid (BR) biosynthesis and signaling pathways (Wang et al. 2025). In the present study, we used transcriptomic and physiological-biochemical analyses to demonstrate that *GhGCS1* promotes lateral root development in cotton by modulating cytokinin (CK) biosynthesis. Our findings revealed that *GhGCS1* enhances resistance to verticillium wilt through the regulation of sphingolipid-associated BR-related and pathogenesis-related (PR) gene expression, rather than through salicylic acid (SA) or methyl jasmonate (JA) signaling pathways. Collectively, our findings demonstrate that precise modulation of sphingolipid metabolism can mitigate the trade-off between plant growth and disease resistance, thereby conferring simultaneous improvements in developmental performance and pathogen defense.

## Results

### *GhGCS1*-overexpression promotes lateral root development in cotton

**Fig. 1:**
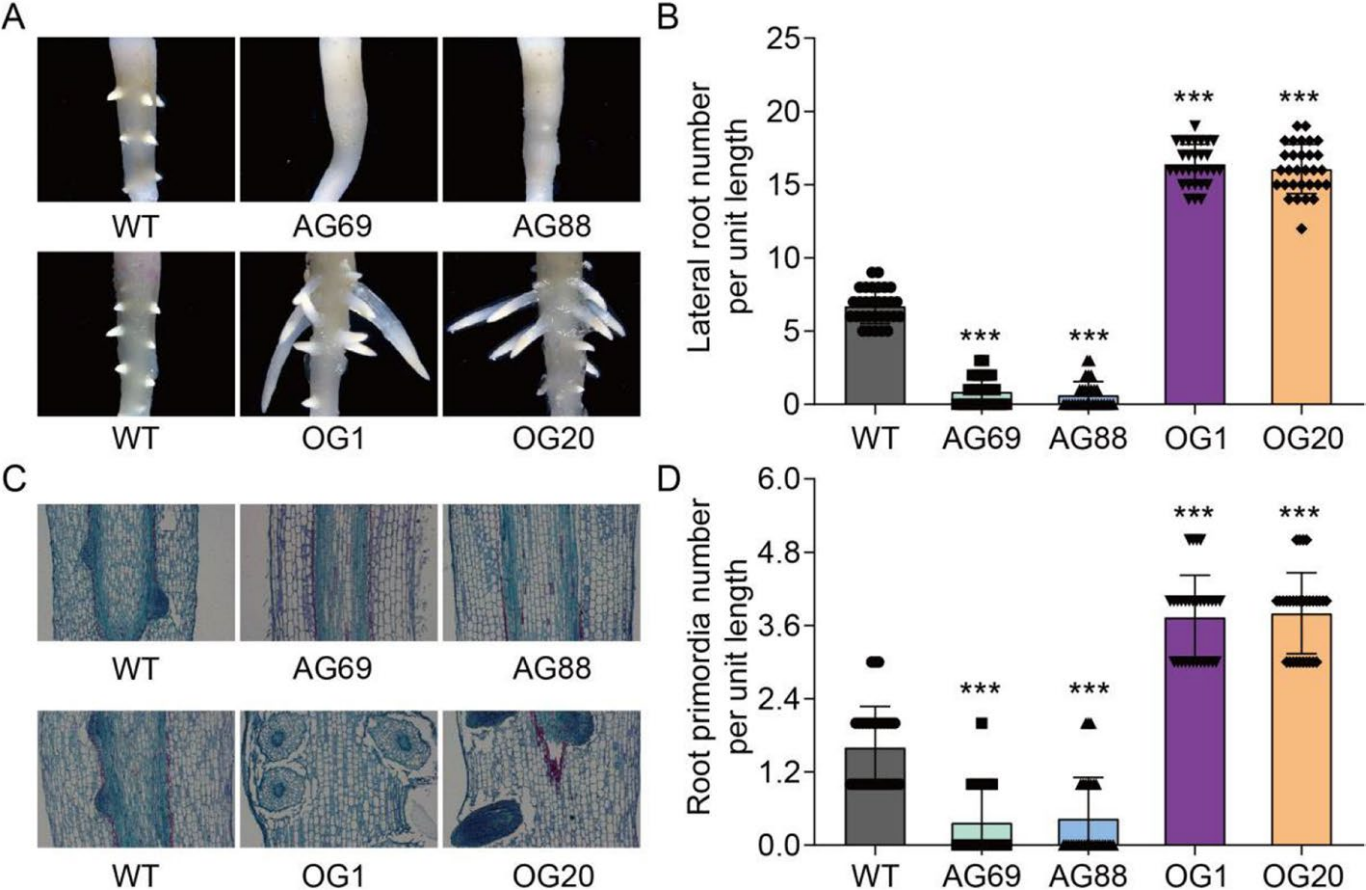
**A** Phenotypes of *GhGCS1* transgenic roots. Images of 5-day roots of *GhGCS1*- antisense (AG69 and AG88) and overexpression (OG1 and OG20) lines. **B** Lateral root number per unit length of *GhGCS1*- antisense and overexpression lines. **C** Longitudinal section of 5-day *GhGCS1*- antisense and overexpression roots. **D** Root primordia number of 5-day *GhGCS1*- antisense and overexpression lines. Error bars, ± SEM. Each analysis was repeated with thirty biological replicates. All *P*-values are based on two-tailed t-tests. ***, *P* < 0.001

Previously, we reported that *GhGCS1* promotes cotton fiber elongation by modulating BR biosynthesis and signaling pathways (Wang et al. 2025). During the cultivation of *GhGCS1* transgenic lines, we observed that *GhGCS1* also affects root development in cotton. Analysis of *GhGCS1* expression revealed comparable expression levels in the roots and fibers of transgenic lines (Supplementary Figure S1, Wang et al. 2025). To further validate the involvement of *GhGCS1* in cotton root development, aseptic seedlings of *GhGCS1* transgenic lines were cultured, and root phenotypes were evaluated five days after germination. The results showed that *GhGCS1* overexpression enhanced lateral root development, whereas antisense-mediated suppression of *GhGCS1* significantly inhibited lateral root outgrowth (Fig. 1A). Consistently, the number of lateral roots per unit primary root length was significantly higher in *GhGCS1-*overexpression lines than in wild-type (WT) plants, whereas antisense suppression lines exhibited a significant reduction (Fig. 1B). Analysis of longitudinal root sections further revealed that the number of lateral root primordia was significantly increased in overexpression lines and decreased in antisense suppression lines relative to WT controls (Fig. 1C and D). Collectively, these results indicate that *GhGCS1* plays a positive regulatory role in promoting lateral root development in cotton.

### Identification of deferentially expressed genes in *GhGCS1*-antisense lines

**Fig. 2:**
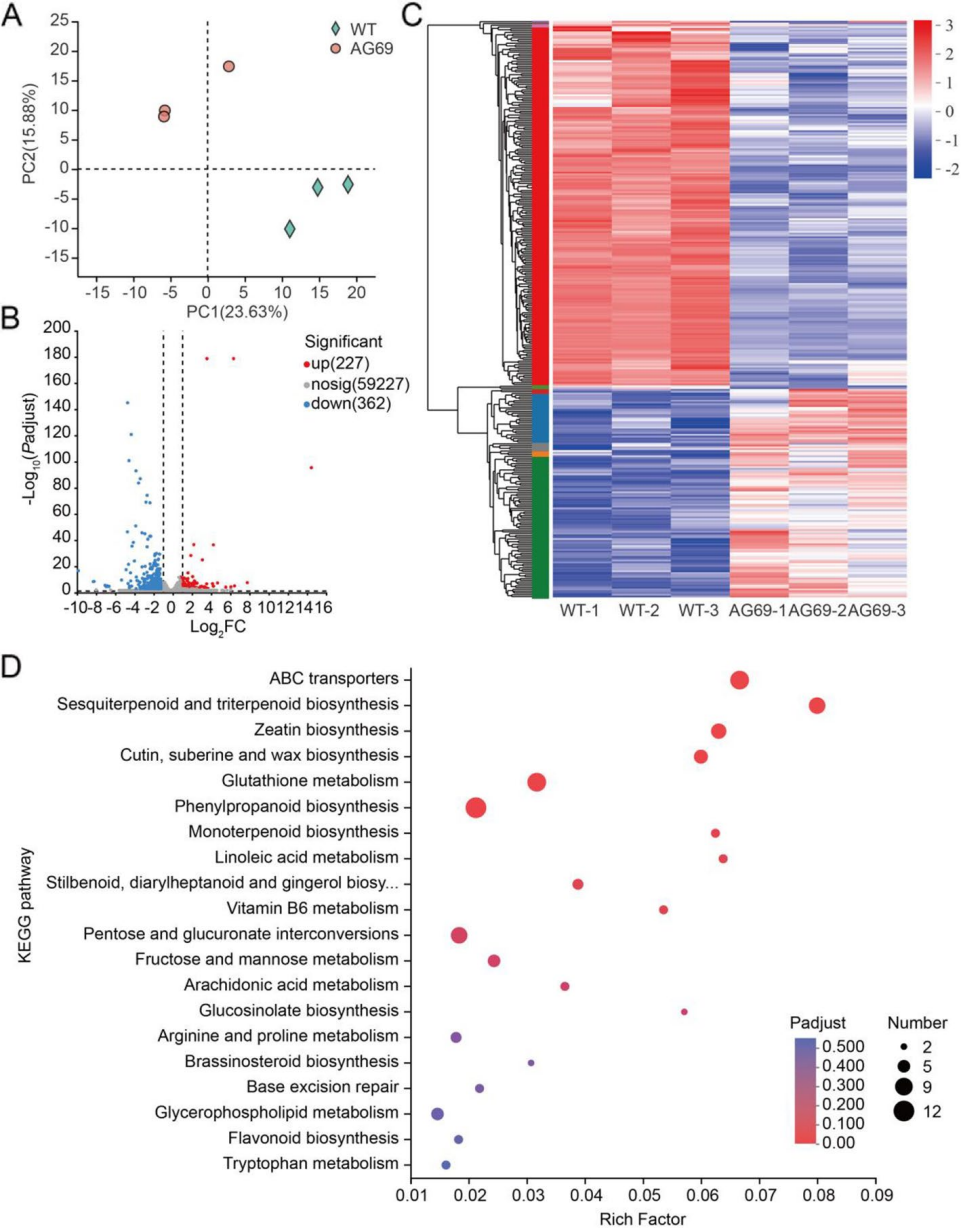
Transcriptome analysis of *GhGCS1* antisense roots. **A** Principal component analysis of the *GhGCS1* antisense root RNA-Seq data. **B** The volcano diagram of differentially expressed genes (DEGs) in *GhGCS1* antisense root. **C** Heat-map hierarchical clustering the DEGs identified in *GhGCS1* antisense root. Each column in the chart represents a replicate of one sample, each row represents a gene, and the color in the chart represents the expression value of the gene after standardized treatment in each sample. Red represents the higher expression level of the gene in the sample, and blue represents the lower expression level. For the specific change trend of the expression level, please see the digital annotation under the color bar on the upper left. On the left are the tree diagram of gene cluster and the module diagram of sub-cluster. **D** The scatter plot shows detailed descriptions of the pathway within the top 20 KEGG enrichment analysis of the 589 DEGs in *GhGCS1*-antisene roots. KEGG, Kyoto Encyclopedia of Genes and Genomes. Analyses of the KEGG enrichment were based on the condition (log2FC > 1, FDR < 0.05). The vertical axis represents the KEGG pathway, and the horizontal axis represents the ratio of the Rich factor (Sample number of genes enriched in the KEGG pathway) to the annotated gene number. The larger the Rich factor, the greater the degree of enrichment, the size of the dots indicates the number of genes in this KEGG pathway, and the color of the dots corresponds to different *P*adjust ranges

To further elucidate the molecular mechanisms underlying *GhGCS1*-mediated regulation of root development in cotton, we conducted a comprehensive RNA-Seq analysis of the *GhGCS1*-antisense line AG69 to generate transcriptome profiles. Each library yielded an average of over 44 million clean reads, providing sufficient depth for accurate quantification of gene expression. Quality control metrics further confirmed the reliability of sequencing data with Q20 and Q30 values exceeding 97% and 93%, respectively (Supplementary Table S1), indicating minimal sequencing errors. Alignment analysis demonstrated high mapping efficiency, with both total and uniquely mapping reads exceeding 93% and 88%, respectively (Supplementary Table S2). Principal component analysis revealed clear separation between the six samples to two distinct groups, corresponding to AG69 and the WT (Fig. 2A). All these data emphasized the reproducibility and reliability of the experimental data.

To identify genes associated with *GhGCS1*-antisense, we performed a systematic pairwise comparison between AG69 and the WT. Through this rigorous analysis, we obtained 589 deferentially expressed genes (DEGs) in the *GhGCS1*-antisense line AG69 compared with the WT. Among these identified DEGs, we obtained 227 up-regulated and 362 down-regulated genes in AG69 (Fig. 2B). To further explore their expression patterns, we generated an expression heat-map of these DEGs, which revealed three distinct sub-clusters. Notably, genes within the red sub-cluster were down-regulated in AG69, whereas, those in the blue and green sub-cluster were largely up-regulated (Fig. 2C).

### Gene Ontology and Kyoto Encyclopedia of Genes and Genomes analysis for DEGs in *GhGCS1*-antisense lines

To gain insight into the potential functions of the 589 DEGs, we performed comprehensive Gene Ontology (GO) and Kyoto Encyclopedia of Genes and Genomes (KEGG) enrichment analyses using the free online tools of Majorbio Cloud Platform (www.majorbio.com). GO analysis revealed that these DEGs were predominantly enriched in several biological processes and molecular functions, including deoxygenase activity, ion homeostasis, and oxidoreductase activity (Figure S2). KEGG pathway analysis further showed that the common DEGs were predominantly associated with pathways such as ABC transporters, zeatin biosynthesis, and glutathione metabolism (Fig. 2D). Among these, the zeatin biosynthesis pathway was of particular interest, because elevated CK levels are known to promote shoot growth while inhibiting root growth (Ma et al. 2024a, b).

### Weighted gene co-expression network analysis with lateral root number

**Fig. 3:**
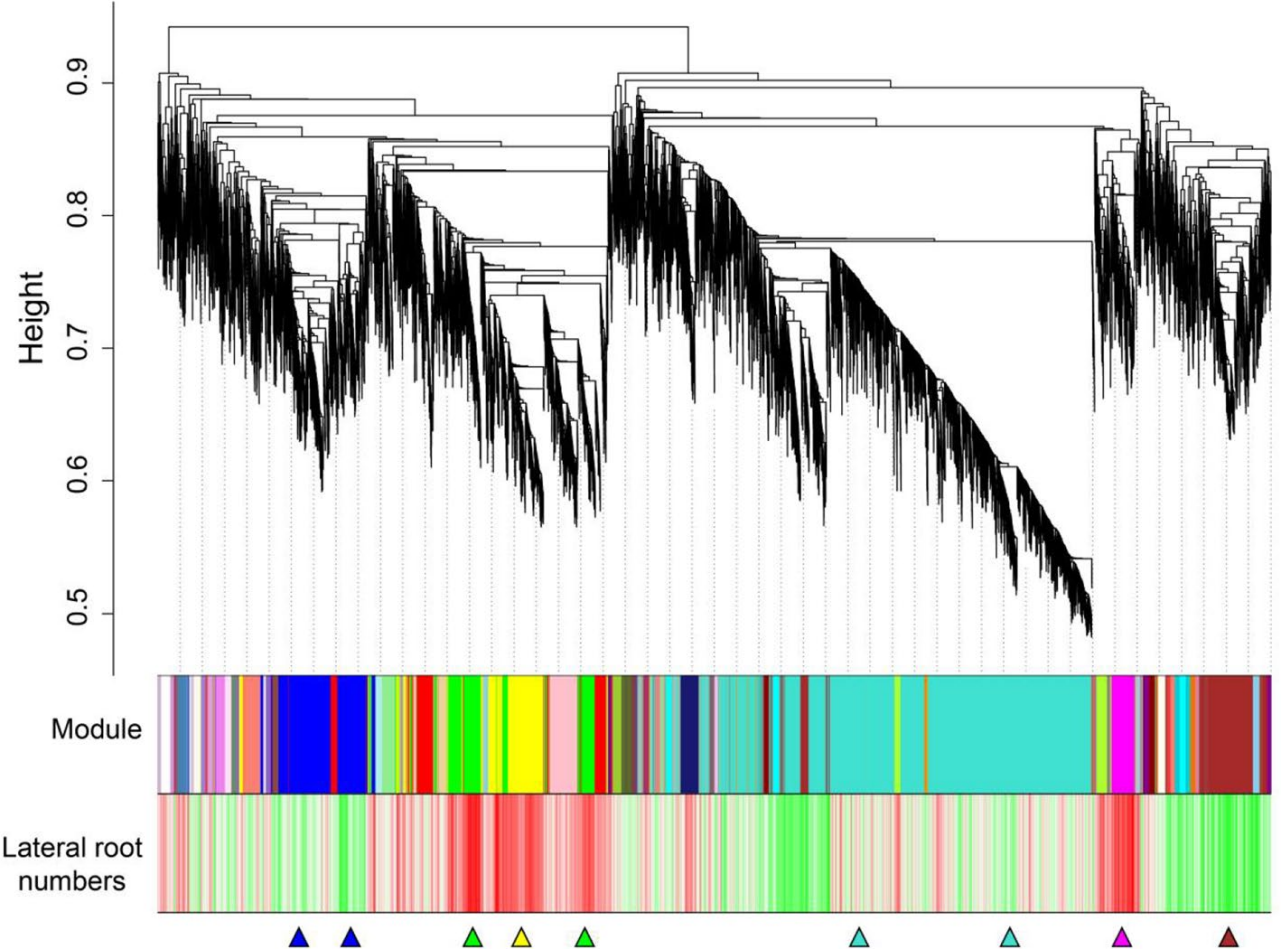
Weighted gene co-expression network analysis of DEGs. Hierarchical cluster tree showing co-expression modules identified by WGCNA with lateral root numbers of *GhGCS1* transgenic lines. Each leaf in the tree represents one gene. The major tree branches constitute 6 modules of total 102 modules, labeled with different colors, including Blue, Green, Yellow, Turquoise, Brown, and Magenta. Red color represents the positive correlation between genes and traits; Green color represents the negative relationships between genes and traits. The blue, green, yellow, turquoise, brown, and magenta triangle denotes the module of particular interest

To identify genes closely associated with root development, we performed weighted gene co-expression network analysis (WGCNA) integrating transcriptomic data and lateral root numbers. From a total of 85,752 genes, 21,255 were filtered and used for WGCNA. Co-expression networks were constructed based on pairwise correlations between gene expression levels and lateral root numbers across all samples. In this context, modules were defined as clusters of highly interconnected genes, with genes within the same cluster exhibiting strong correlation coefficients. Through this analytical approach, we identified 102 distinct modules, each labeled with a unique color (Fig. 3). Of these, six modules blue, green, yellow, turquoise, brown, and magenta were of particular interest. The green, yellow, and magenta modules contained 2294 genes, whose expression was positively correlated with lateral root number, whereas the blue, turquoise, and brown modules included 7485 genes that were negatively correlated. Notably, the CK biosynthetic gene *GhCYP735A* was present in the negatively correlated modules consistent with the reduced lateral root phenotype observed in *GhGCS1*-antisense lines.

### *GhGCS1*-overexpression inhibits CK biosynthesis in cotton roots

**Fig. 4:**
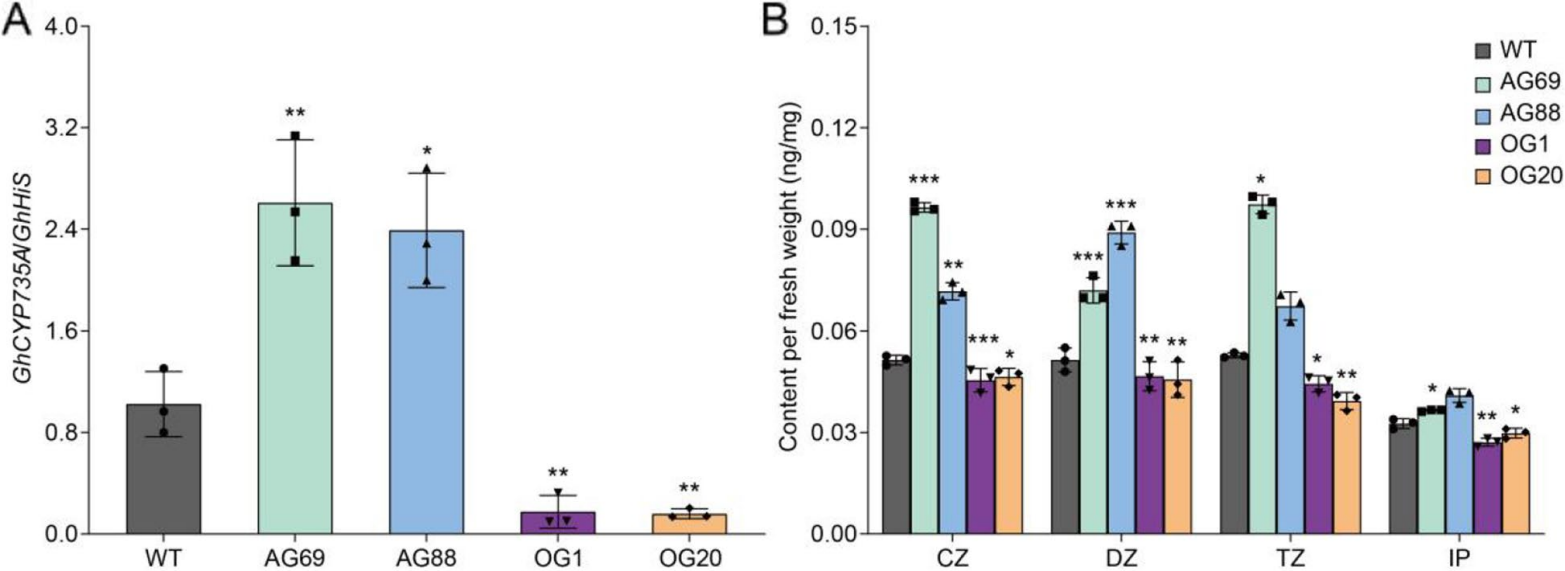
*GhGCS1* inhibits the biosynthesis of cytokinin in roots. **A** The expression of cytokinin biosynthetic gene *GhCYP735A* in *GhGCS1* transgenic roots detected by qRT-PCR. **B** The amounts of cytokinin in *GhGCS1* transgenic roots. CZ, cis-Zeatin; DZ, Dihydrozeatin; TZ, trans-Zeatin; IP, Isopentenyladenine. Error bars, ± SEM. Each analysis was repeated with three biological replicates. All *P*-values are based on two-tailed t-tests. *, *P* < 0.05; **, *P* < 0.01; ***, *P* < 0.001

To validate the transcriptomic findings for *GhCYP735A*, we examined its expression in *GhGCS1* transgenic lines using quantitative real-time PCR (qRT-PCR). Consistent with the RNA-seq data, *GhCYP735A* was significantly up-regulated in *GhGCS1*-antisense lines and strongly repressed in its overexpression lines (Fig. 4A). Subsequently, we measured CK levels in the roots of *GhGCS1* transgenic plants. Isopentenyladenine (IP) levels were slightly elevated in the roots of *GhGCS1*-antisense lines, and modestly reduced in the roots of its overexpression lines. Furthermore, the levels of cis-Zeatin (CZ), Dihydrozeatin (DZ), and trans-Zeatin (TZ) were significantly increased in the roots of *GhGCS1*-antisense lines, but markedly decreased in the roots of its overexpression lines (Fig. 4B). We also analyzed the expression of auxin transport-related genes. The auxin transporter genes *GhAUX1* and *GhPIN2* were down-regulated in *GhGCS1*-antisense lines, but strongly up-regulated in its overexpression lines, while expression levels of auxin biosynthesis and signaling genes remained largely unchanged (Figure S3). These results suggest that *GhGCS1* may play a regulatory role in root development by modulating auxin transport, in addition to suppressing CK biosynthesis, consistent with the observed root phenotypes in the respective transgenic lines.

### *GhGCS1* overexpression enhances cotton resistance to verticillium wilt

**Fig. 5:**
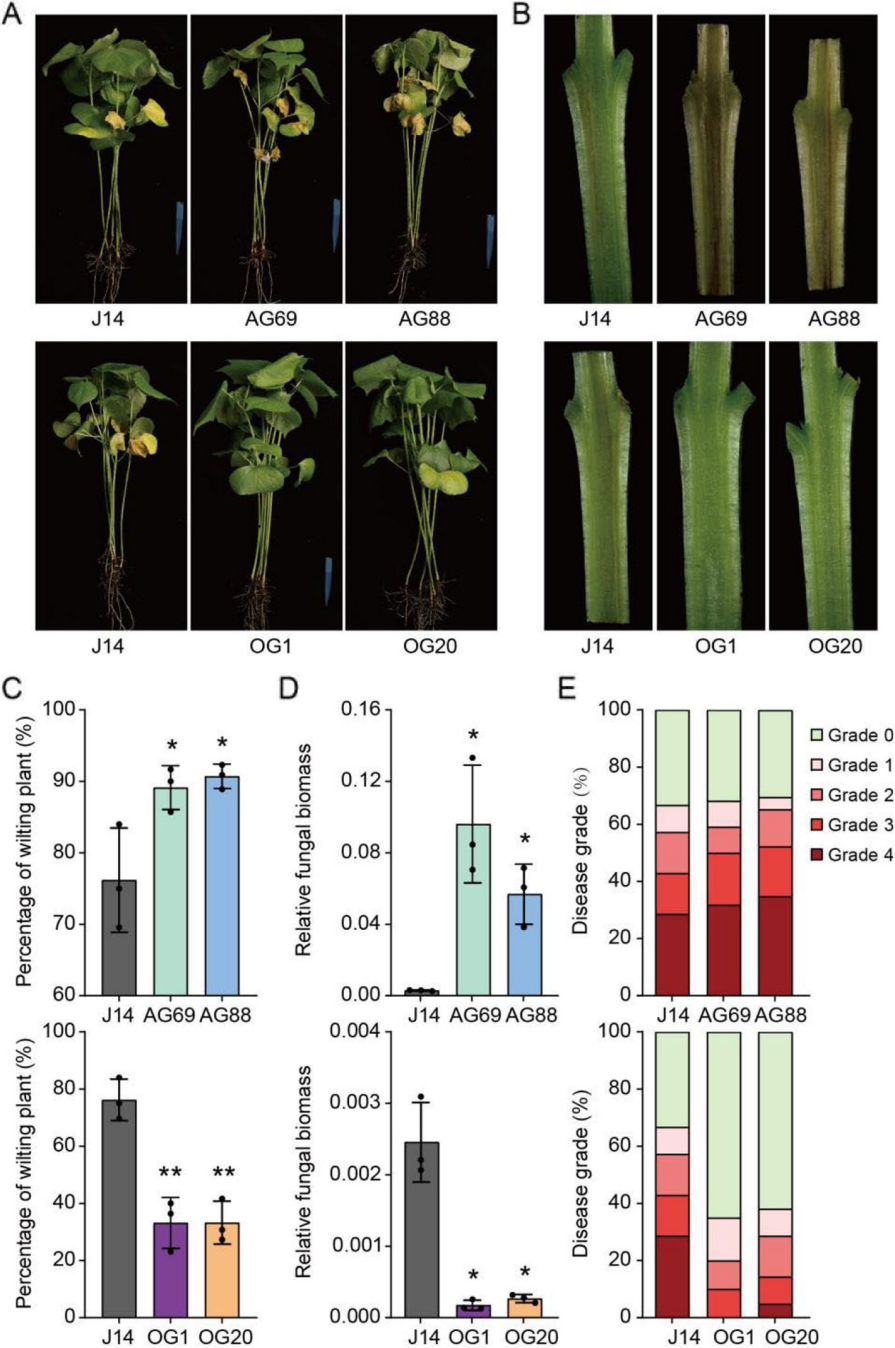
*GhGCS1* enhances cotton verticillium wilt resistance. **A**, **B** Phenotypes (**A**), stem longitudinal section (**B**) of *GhGCS1* transgenic line after 7-day *V. dahliae* treatments. **C**–**E** Wilting percentage (**C**), the amounts of *V. dahliae* (**D**), and disease grade of *GhGCS1* transgenic line after 7-day *V. dahliae* treatments. Error bars, ± SEM. Each analysis was performed in at least three biological replicates. All *P*-values are based on two-tailed t-tests. *, *P* < 0.05; **, *P* < 0.01

In previous studies, we showed that the sphingolipid synthesis inhibitor FB1, a metabolite and toxin produced by *V. dahliae*, induces verticillium wilt by disrupting sphingolipid biosynthesis in plants (Xu et al. 2022). However, the role of sphingolipids in cotton defense against this pathogen remains to be elucidated. To investigate this, we evaluated the responses of *GhGCS1* transgenic cotton lines to *V. dahliae* infection. Two weeks post inoculation, WT plants exhibited typical disease symptoms, including defoliation, necrosis, and wilting. Symptoms were more severe in *GhGCS1*-antisense lines, whereas its overexpession lines displayed only mild symptoms (Fig. 5A and S3). Consistent with these observations, stem cross-sections of WT plants showed brown discoloration of the xylem, which was more pronounced in *GhGCS1*-antisense lines. In contrast, *GhGCS1*-overexpression lines exhibited significantly reduced xylem browning, with some individuals showing almost no visible discoloration (Fig. 5B).

The wilting rate of WT cotton was approximately 75%, whereas it reached 90% in *GhGCS1*-antisense lines, and was only 30% in its overexpression lines (Fig. 5C). To further assess pathogen colonization, we quantified the relative biomass of *V. dahliae* in *GhGCS1* transgenic lines using qRT-PCR. The relative fungal biomass was approximately 0.0025 in WT plants, exceeded 0.05 in *GhGCS1*-antisense lines, and was nearly undetectable in its overexpression lines (Fig. 5D). Consistently, the disease index of WT plants was approximately 66, increased to over 70 in *GhGCS1*-antisense lines and decreased to below 40 in its overexpression lines (Fig. 5E). Collectively, these results demonstrate that overexpression of *GhGCS1* enhances cotton resistance to verticillium wilt.

### *GhGCS1* enhances cotton resistance to verticillium wilt through BR-related and PR pathways

**Fig. 6:**
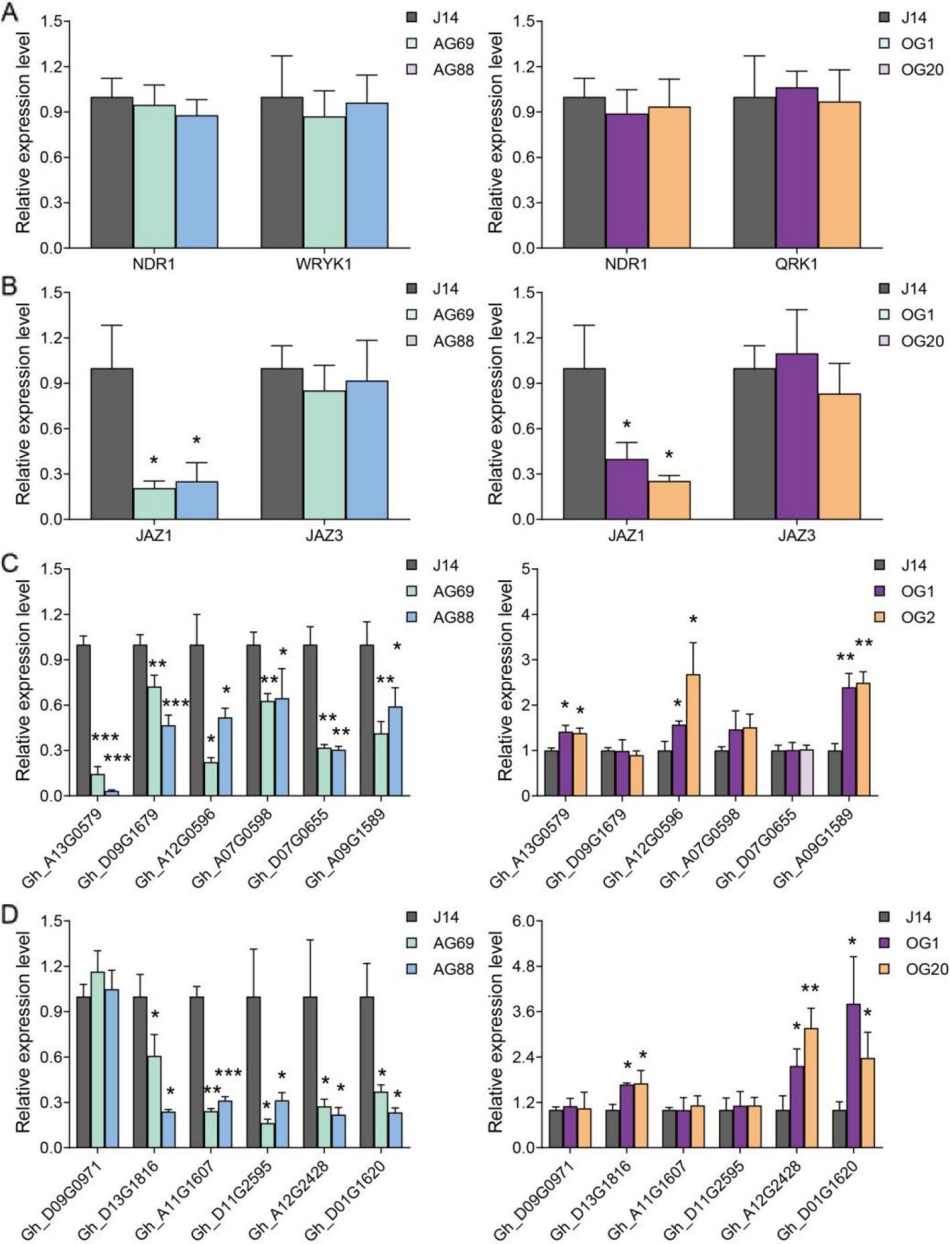
*GhGCS1* enhances cotton verticillium wilt resistance through the BR and PR signaling pathways. **A**, **B** The expression level of SA- (**A**) and JA- (**B**) related genes in *GhGCS1* transgenic roots after 7-day *V. dahliae* treatment detected by qRT-PCR. **C**, **D** The expression level of BR- (**C**) and PR- (**D**) related genes in *GhGCS1* transgenic roots after 7-day *V. dahliae* treatment detected by qRT-PCR. Error bars, ± SEM. Each analysis was repeated with three biological replicates. All *P*-values are based on two-tailed t-tests. *, *P* < 0.05; **, *P* < 0.01; ***, *P* < 0.001

To further elucidated the molecular mechanisms by which *GhGCS1* enhances resistance to verticillium wilt in cotton, we examined the expression patterns of key disease-related genes in *GhGCS1* transgenic lines following *V. dahliae* infection. After *V. dahliae* treatment, the expression levels of *NDR1* and *WRKY1*, two genes associated with the SA signaling pathway, showed no significant differences between WT plants and *GhGCS1*- antisense or overexpression lines (Fig. 6A). Similarly, the expression of *JAZ3*, a JA signaling-related gene, remained unchanged,where as *JAZ1* expression was significantly downregulated in both *GhGCS1*- antisense and overexpression lines (Fig. 6B). Collectively, these results indicate that *GhGCS1* is unlikely to regulate cotton resistance to verticillium wilt via the SA or JA signaling pathways.

In our previous study, we found that both FB1 treatment and *V. dahliae* infection altered the expression of BR-related and PR genes (Xu et al. 2022). Accordingly, we assessed the expression levels of BR-related and PR genes in *GhGCS1* transgenic cotton lines following two weeks *V. dahliae* infection. The results showed that the expression of BR-related genes, including *GhBAK1* (*Gh_A13G0579*), *Gh_D09G1679*, *Gh_A12G0596*, *Gh_A07G0598*, *Gh_D07G0655*, and *Gh_A09G1589*, was down-regulated in *GhGCS1*-antisense lines. In contrast, all of these genes were up-regulated in *GhGCS1*-overexpression lines, with the exception of *Gh_D07G0655* (Fig. 6C). Regarding the PR genes, *Gh_D09G0971* exhibited no significant changes across the *GhGCS1* transgenic lines. The expression levels of *Gh_D13G1816*, *Gh_A11G1607*, *Gh_D11G2595*, *Gh_A12G2428*, and *Gh_D01G1620* were down-regulated in *GhGCS1*-antisense lines. In *GhGCS1*-overexpression lines, all of these genes, except *Gh_A11G1607* and *Gh_D11G2595,* were up-regulated (Fig. 6D). These results are consistent with our previous findings (Xu et al. 2022), and suggest that *GhGCS1* enhances cotton resistance to verticillium wilt by modulating BR-related and a subset of PR gene.

## Discussion

### GCS promotes cotton root development by inhibiting CK biosynthesis

Sphingolipids have long been recognized as structural components of cellular membranes. However, increasing evidence indicates that they also function as bioactive signaling molecules regulating multiple aspects of plant development (Liu et al. 2021; Yang et al. 2025b). FB1, as a specific inhibitor of sphingolipid biosynthesis, is widely used to elucidate the functional roles of sphingolipids in plant growth and development (Zeng et al. 2020). Transcriptomic and proteomic analyses of FB1-treated cotton ovules have shown that FB1 blocked cotton fiber cell elongation by disrupting phenylpropanoid and flavonoid biosynthesis pathways (Wang et al. 2020). *GhIQD10*, a gene significantly downregulated by FB1 treatment, modulates calcium signaling during fiber development through its interaction with GhCaM7, thereby influencing fiber elongation. Functional analysis demonstrated that overexpression of *GhIQD10* results in a short-fiber phenotype, further confirming its regulatory role in fiber development (Xu et al. 2023). Another FB1 responsive gene, *GhMYB86*, has been identified as a key regulator of fiber elongation. GhMYB86 regulates microtubule organization at the fiber tip by modulating the expression of *GhTUB7*, a tubulin gene essential for cytoskeletal dynamics. These findings provide compelling evidence for the interplay between membrane components and the cytoskeleton during plant cell elongation (Xu et al. 2024). Additionally, several sphingolipid biosynthesis-related genes, including *GhCS1*, *GhKCRL1*, *GhLCBK1*, and *GhGCS1*, have been shown to influence cotton fiber development (Li et al. 2022; Meng et al. 2022; Zhang et al. 2024; Wang et al. 2025). Consistently, mutations in sphingolipid biosynthetic genes, such as *SBHs* in Arabidopsis and *IPCSs* in rice, lead to dwarf phenotypes (Chen et al. 2008; Wang et al. 2023), highlighting the conserved role of sphingolipids in regulating plant growth across species.

Although the role of sphingolipids in root development remains poorly understood, previous studies have shown that inhibitors of sphingolipid biosynthesis can suppress root growth (Krüger et al. 2013; Rugen et al. 2018; Altamura et al. 2023). In the present study, we demonstrated that overexpression of *GhGCS1* promotes lateral root formation in cotton, whereas suppression of *GhGCS1* expression inhibits this process. Transcriptomic analyses suggested that *GhGCS1* regulates lateral root development by modulating CK biosynthesis. This hypothesis was further supported by measurements of endogenous CK levels, which revealed significant differences between *GhGCS1*- overexpression and antisense plants. Collectively, these findings indicate that *GhGCS1* promotes cotton root development by influencing CK synthesis, thereby providing novel insights into the role of sphingolipids in regulating plant growth and development, particularly root architecture. Moreover, our results highlight the multifaceted functions of sphingolipids beyond their structural roles, positioning them as key regulators of plant developmental processes.

### GCS enhances cotton resistance to verticillium wilt through the BR and PR signaling pathways

Sphingolipids not only play essential structural and functional roles in plant growth and development, but also act as key signaling molecules in plant stress responses (Ali et al. 2018; Huby et al. 2019). Specific sphingolipid species confer protection against various abiotic stresse. For instance, plant Δ8 sphingolipid desaturases significantly enhance resistance to aluminum toxicity and low-temperature stress (Ryan et al. 2007; Chen et al. 2011). Notably, GIPCs function as Na^+^ receptors in plants; direct Na^+^ binding by GIPCs represents a key step in sodium sensing that triggers calcium-dependent signaling cascades, ultimately enhancing salt tolerance, and enabling plant survival under saline conditions (Jiang et al. 2019). Moreover, sphingolipid remodeling within the PM has been identified as a key adaptive mechanism contributing to osmotic stress tolerance in Arabidopsis thaliana (Li et al. 2025b). The PHR1-NPC4-GCS regulatory module further fine-tunes sphingolipid homeostasis, facilitating plant adaptation to phosphate deficiency and supporting sustained growth (Yang et al. 2025a).

Regarding biotic stress resistance, GIPCs function as receptors for the NLP toxin produced by *V. dahliae*, and alterations in GIPC composition confer enhanced resistance to NLP-induced toxicity (Lenarčič et al. 2017). Phytosphingosine has been shown to regulate PD permeability by promoting of PDLP5-mediated callose deposition, thereby enhancing resistance to both fungal (*V. dahliae*) and bacterial (*Pseudomonas syringae* pv. tomato DC3000) pathogens (Liu et al. 2020). Additionally, C1P strengthens defense responses against *S. sclerotiorum* in *Brassica napus* (Ouyang et al. 2025). As described above, *GhGCS1* promotes root development in cotton. Given that *V. dahliae* is a widespread soil-borne pathogen causing vascular wilt in more than 200 plant species (Li et al. 2020), investigating the potential role of *GhGCS1* in modulating cotton resistance to this economically significant disease is important. Notably, in the present study, antisense-mediated silencing of *GhGCS1* resulted in markedly increased susceptibility to *V. dahliae*, whereas overexpression of *GhGCS1* significantly enhanced cotton resistance to verticillium wilt. Further molecular mechanistic analyses revealed that *GhGCS1*, a key enzyme in sphingolipid biosynthesis, enhances disease resistance by modulating the expression of sphingolipid-associated BR-related and PR genes. Together, these findings expand our understanding of the role of sphingolipids in cotton resistance to verticillium wilt and provide valuable genetic resources and potential strategies for improving disease resistance in cotton breeding programs.

### Precise regulation of sphingolipid biosynthesis contributes to maintaining the trade-off between plant development and disease resistance

Plant yield and stress resistance are governed by intricate molecular regulatory networks. These traits are often antagonistic, giving rise to the so-called “growth-defense trade-off” phenomenon, in which plants preferentially allocate limited resources to defense responses at the expense of growth when challenged by biotic or abiotic stresses. Consequently, genetic variants that enhance stress resistance frequently compromise plant growth, whereas variants that promote growth may reduce resistance (Derbyshire et al. 2024). In recent years, plant breeding efforts have increasingly focused on achieving a balanced regulation between growth and stress resistance, with the goal of developing crop varieties that maintain stable or even enhanced yields under adverse environmental conditions. Several studies have demonstrated that precise regulatory mechanisms can mitigate this trade-off. For example, reversible phosphorylation of IPA1 enables plants to rapidly activate defense responses against fungal pathogens, and subsequently reallocate resources back to growth within a few days, thereby maintaining both pathogen resistance and crop yield (Wang et al. 2018). In *Arabidopsis*, the homeobox transcription factor HB34 suppresses JA biosynthesis while promoting the expression of growth-related genes, contributing to a balanced coordination between immunity and growth (Li et al. 2025a). Additionally, targeted insertion of heat-shock cis-regulatory elements into cell wall invertase genes using prime editing improves carbon partitioning, resulting in climate-smart crops with increased yields under normal conditions and maintained yield stability under heat stress in both tomato and rice (Lou et al. 2025).

In our previous studies, we demonstrated that overexpression of *GhIQD10* enhances cotton resistance to verticillium wilt (Xu et al. 2022). However, this genetic modification is accompanied by the development of a short-fiber phenotype (Xu et al. 2023), indicating that sphingolipids and their associated genes play a pivotal role in coordinating cotton growth and disease resistance. In subsequent research on *GhGCS1*, we found that its overexpression not only promotes fiber elongation (Wang et al. 2025) and root development but also significantly enhances resistance to verticillium wilt (this study). Collectively, these findings suggest that precise modulation of the sphingolipid biosynthesis pathway can effectively reconcile the trade-off between cotton growth performance and stress tolerance. This discovery provides a novel strategy for simultaneously improving plant stress resistance and developmental traits, offering both a theoretical framework and practical potential for advancing crop genetic improvement.

## Conclusion

**Fig. 7:**
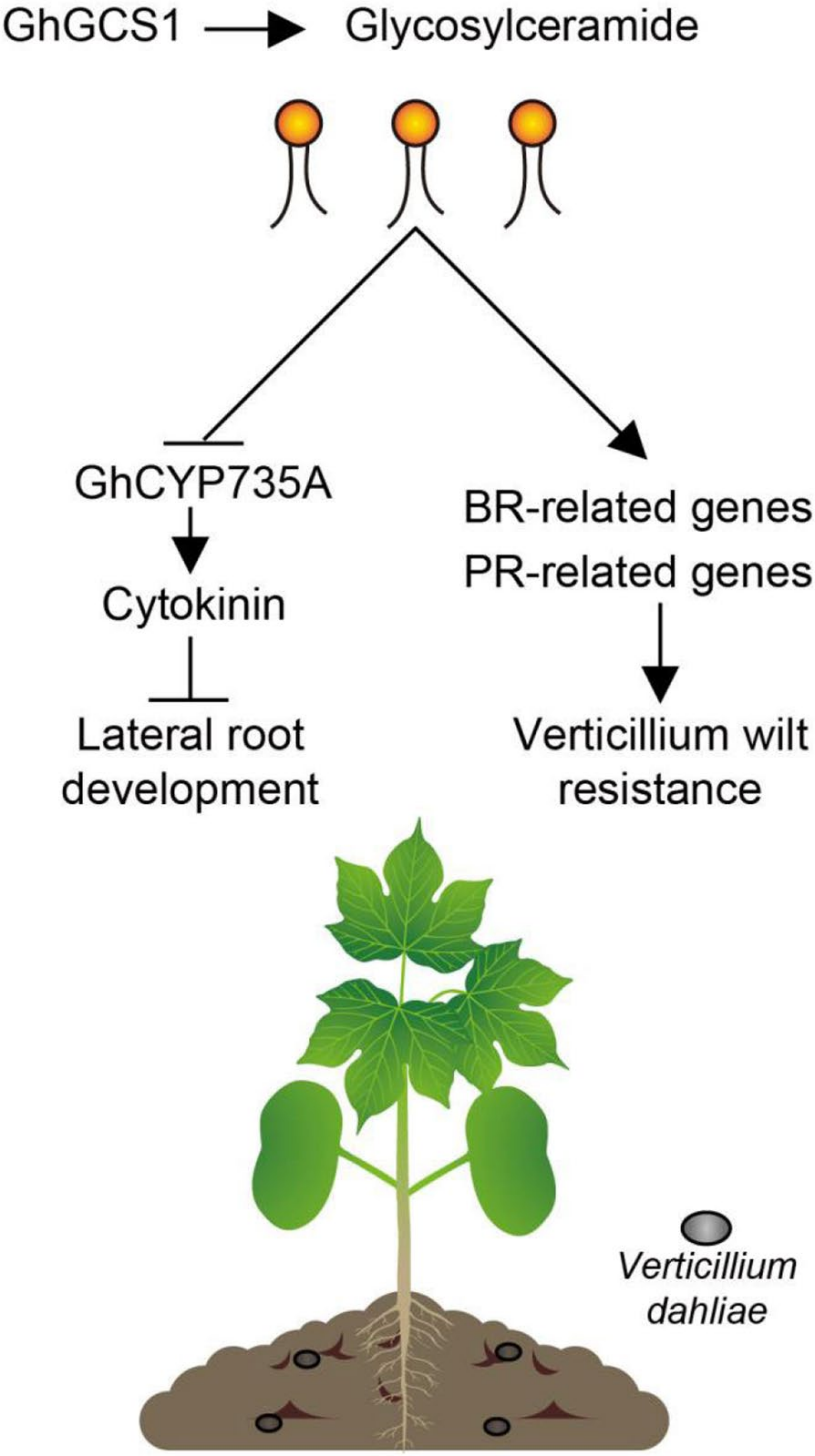
Proposed working model of *GhGCS1* in root development and verticillium wilt resistance. In the one hand, In the one hand, overexpression of the *GhGCS1* gene promotes the accumulation of glycosylceramide, which subsequently promotes lateral root development in cotton by suppressing the expression of *GhCYP735A*, thereby limiting cytokinin synthesis in roots. On the other hand, it enhances cotton verticillium wilt resistance by up-regulating sphingolipid-associated BR-related and PR-related genes

In this study, we demonstrate that *GhGCS1* promotes cotton root development by inhibiting CK biosynthesis while simultaneously enhancing resistance to verticillium wilt via the upregulation of BR-related and PR genes. These findings indicate that precise regulation of sphingolipid metabolism can effectively balance the trade-off between plant growth and defense (Fig. 7). Overexpression of *GhGCS1* resulted in the development of cotton lines with improved root architecture, enhanced fiber quality, and increased disease resistance. Together, these results provide a strong theoretical framework and valuable genetic resources for breeding high-quality, high-yielding, and stress-tolerant cotton varieties.

## Materials and methods

### Materials and growth conditions

WT Jimian 14 (*Gossypium hirsutum* L. Jimian14) was provided by Professor Ma Zhiying (Hebei Agricultural University). The *GhGCS1*-antisense lines (AG69 and AG88) and overexpression lines (OG1 and OG20) were generated previously and are maintained at the Biotechnology Research Center of Southwest University (Wang et al. 2025). All plants were grown under natural conditions in the experimental field of the College of Agronomy and Biotechnology, Southwest University, Chongqing, China. The highly virulent *V. dahliae* strain V991 used for inoculation were routinely grown in potato dextrose broth (PDB) at 26 °C.

### Histological examination

To observe root phenotypes at the seedling stage, cotton seeds were surface-sterilized with 75% ethanol and subsequently planted on 1/2 Murashige and Skoog medium. Seedlings were grown at 26 ℃ under long-day conditions (16 h light/8 h dark) with 125 mmol m^–2^s^–1^ white light for five days, after which root images were captured. Root segments of uniform position and length (1 cm from the root base) were excised and fixed in FAA solution (37% formaldehyde/glacial acetic acid/50% ethanol, 5:6:89, by volume) for 12 h at 25 °C. Following gradient dehydration and infiltration, samples were embedded in paraffin and 8-μm sections were generated. Sections were stained with 1% solid green and examined under a microscope (Olympus BX 51), with representative images photographed for analysis.

### Transcriptome analysis

Roots of 16 seedlings of each transgenic lines were collected after 5-day growth with three biological replicates. To ensure robust data collection, we meticulously collected sixteen random roots of AG69 and WT. The samples were stored at −80 ℃ at the College of Agronomy and Biotechnology, Southwest University. RNA-Seq analysis was performed by Shanghai Majorbio Bio-pharm Technology Co., Ltd. following the previously described protocol (Xu et al. 2023) with some modifications. Briefly, The mRNA was enriched using the oligo(dT) magnetic beads. The sample libraries were qualified and quantified by Agilent 2100 Bioanaylzer and Nanodrop2000. The library products were sequenced via Illumina Novaseq 6000. The raw data were filtered with the FASTQ_Quality_Filter tool from the FASTX-toolkit (http://hannonlab.cshl.edu/fastx_toolkit/). And then were aligned to the *G. hirsutum* genome (https://phytozome.jgi.doe.gov/pz/portal.html#!info?alias=Org_Ghirsutum_er) using HISAT2 (http://ccb.jhu.edu/software/hisat2/index.shtml). Transcriptome data were analyzed on the free online platform, Majorbio Cloud Platform (www.majorbio.com). The criteria for defining DEGs included a two-fold expression (AG69/WT) difference coupled with a false discovery rate (FDR) value of less than 0.001. And Up-regulated genes are defined as those with Log2 (fold change) > 1, while down-regulated genes are defined as those with Log2 (fold change) < –1.

### GO and KEGG analysis for DEGs

To gain insights into the biological functions of the DEGs, GO (http://www.geneontology.org) and KEGG (http://www.kegg.jp/kegg/kegg1.html) enrichment analyses were performed using the free online tools of the Majorbio Cloud Platform (www.majorbio.com).

### Weighted gene co-expression network analysis

WGCNA was performed using the Majorbio Cloud Platform, a free online platform. A total of 21,255 genes were selected after filtering out genes with a mean TPM value less than 1 and a coefficient of variation less than 0.1. These genes were further utilized for co-expression network construction using the WGCNA R package (version 1.63; https://cran.r-project.org/web/packages/WGCNA/index.html). Modules were identified using the following parameters: a soft-thresholding power (β) of 8, a minimum module size of 30, and a merge cut height of 0.25. Gene significance values were calculated using Spearman's correlation to evaluate the correlation between gene expression and lateral root number.

### RNA extraction and qRT-PCR

Total RNA was extracted from 16 seedlings, with or without *V. dahliae* inoculation, using the Plant Total RNA Extraction Kit (Tiangen, China), according to the manufacturer’s instructions. First-strand cDNA was synthesized from 2 µg of total RNA using the All-in-one 5 × RT MasterMix (abm, China), following the manufacturer’s protocol. Gene-specific primers are listed in Supplementary Table S3. qRT-PCR was performed on a CFX96™ Optical Reaction Module (Bio-Rad, USA) using 2 × ChamQ SYBR qPCR Master Mix (Vazyme, China), according to the manufacturer’s instructions. GhHis was used as the internal control. All reactions were conducted with three biological replicates.

### CK content detection

CK quantification was performed as previously reported (Gao et al. 2014) and carried out at Nanjing Convinced-Test Technology Co., Ltd. Briefly, CKs were extracted from roots of each transgenic cotton line using the isopropanol/water/hydrochloric acid extraction system. Following extraction, the samples were analyzed using an AB ExionLC/Agilent 1290 high-performance liquid chromatography system coupled with an AB Sciex QTRAP 6500 + mass spectrometer, with quantitative analysis carried out via the internal standard method.

### Detection of plant resistance to *V. dahliae*

For resistance assays, plants were grown in sand for two weeks until two true leaves had fully developed. Then, seedlings were inoculated by dipping the roots into a *V. dahliae* conidial suspension (1 × 10^7^ conidia L^−1^), followed by one week hydroponic cultivation. Verticillium wilt symptoms were subsequently recorded.

For fungal biomass quantification, leaves form three inoculated plants were harvested at 7 days post-inoculation (dpi). Real-time qRT-PCR was performed using the Novostar-SYBR Supermix (Novoprotein, China), according to the manufacturer’s instructions. Primers ITS1-F and ST-VE1-R, specific for the *V. dahliae* internal transcribed spacer region of ribosomal DNA, were used, with *GhHis* serving as the endogenous reference gene (Supplementary Table 3).

For histological observation of mycelial growth, stems from three inoculated plants were collected at 7 dpi, and sectioned longitudinally. Images were captured using a SYCOP3 stereoscope (Zeiss, Germany).

## Supplementary Information

Supplementary Material 1.

## Data Availability

The raw sequence data supporting the results of this article were submitted to the CNCB database (https://www.cncb.ac.cn/), and can be found with BioProject PRJCA043932. Further information and requests for data and material should be directed to and will be fulfilled by Fan Xu (xufanfeiren@163.com) and Ming Luo (luo0424@126.com).

## Abbreviations

BR: Brassinosteroid
PR: Pathogenesis-related
PM: Plasma membrane
LCB: Long-chain base
LCFA: Long-chain fatty acid
VLCFA: Very long-chain fatty acid
RNAi: RNA interference
GA: Gibberellin
ABA: Abscisic acid
*V. Dahliae*: *Verticillium dahliae*
PD: Plasmodesmata (PD)
GIPC: Glycosylinositol phosphorylceramide
GlcCers: Glucosylceramide
Cer: Ceramide
C1P: Ceramide-1-phosphate
FB1: Fumonisin B1
SA: Salicylic acid
JA: Methyl jasmonate
WT: Wild type
DEGs: Differentially expressed gene
FDR: False discovery rate
GO: Gene Ontology
KEGG: Kyoto Encyclopedia of Genes and Genomes
CK: Cytokinin
WGCNA: Weighted gene co-expression network analysis
IP: Isopentenyladenine
CZ: Cis-Zeatin
DZ: Dihydrozeatin
TZ: Trans-Zeatin
