# Supplementary material for: Synergistic role of GhGCS1 in cotton root development and verticillium wilt resistance
Source: Stress Biol. 2026 Feb 6;6(1):11. doi: 10.1007/s44154-026-00293-6 (PMC12881189; doi:10.1007/s44154-026-00293-6)
Supplement: Supplementary file 1 — Supplementary Material 1. [file 44154_2026_293_MOESM1_ESM.docx]

**Supplementary Table S1. Evaluation of sequencing quality**

| Sample | Raw reads | Raw bases | Clean reads | Clean bases | Error rate(%) | Q20(%) | Q30(%) | GC content(%) |
| --- | --- | --- | --- | --- | --- | --- | --- | --- |
| WT_1 | 50863386 | 7680371286 | 49288790 | 7196349928 | 0.026 | 97.64 | 93.25 | 44.46 |
| WT_2 | 48569254 | 7333957354 | 47157984 | 6874081392 | 0.0254 | 97.85 | 93.83 | 44.56 |
| WT_3 | 45797994 | 6915497094 | 44530754 | 6521785744 | 0.0256 | 97.8 | 93.66 | 44.41 |
| AG69_1 | 47980896 | 7245115296 | 46749660 | 6834871805 | 0.0256 | 97.8 | 93.66 | 44.43 |
| AG69_2 | 47497092 | 7172060892 | 46229994 | 6778981033 | 0.0259 | 97.68 | 93.34 | 44.37 |
| AG69_3 | 46960322 | 7091008622 | 45642314 | 6685983782 | 0.026 | 97.65 | 93.28 | 44.57 |

**Supplementary Table S2. Mapping efficiency statistics**

| Sample | Total reads | Total mapped | Multiple mapped | Uniquely mapped |
| --- | --- | --- | --- | --- |
| WT_1 | 49288790 | 46958279(95.27%) | 5373922(10.9%) | 41584357(84.37%) |
| WT_2 | 47157984 | 44968822(95.36%) | 5392704(11.44%) | 39576118(83.92%) |
| WT_3 | 44530754 | 42537857(95.52%) | 4710802(10.58%) | 37827055(84.95%) |
| AG69_1 | 46749660 | 44445758(95.07%) | 5037294(10.78%) | 39408464(84.3%) |
| AG69_2 | 46229994 | 44011308(95.2%) | 4853522(10.5%) | 39157786(84.7%) |
| AG69_3 | 45642314 | 43048534(94.32%) | 4878589(10.69%) | 38169945(83.63%) |

**Supplementary Table S3. List of primers used in this study**

|  | Forward (5'-3') | Reverse (5'-3') |
| --- | --- | --- |
| q*GhHis* | TCCACGATTCTCACCGTTGC | CTACCACTACCATCATGGC |
| q*GhGCS1* | AGAGGGAGGGAAGAGCAGAAG | AGAGGGAGGGAAGAGCAGAAG |
| qGh*NDR1* | TAACTTGACGGTGGCTTCGC | CACTGAAGTCGACAAATCCA |
| qGh*WRKY1* | CAGCTTCAGCCCATATCTTTG | ATCCAACATGCATCATATGCC |
| qGh*JAZ1* | CAGCACCACGGTAACTCAAG | GCTCTCCTTGCAATTGGTAG |
| qGh*JAZ3* | GCGGGTGAAGTGAATGTCTTTGAT | GAACTGGTTTCAAAATAGGAGTCTGGA |
| q*Gh_A13G0579* | CCAACCGATACCAACCCTTG | AGCATCCCCAGGTCTGGAAC |
| q*Gh_A07G0598* | AAGTTGACGGCAGCTAGCAC | AAGCTCCTTTGCCTCCTCTG |
| q*Gh_D05G3727* | CCTTGTAGGGTGGGCAAAGC | CATGGTCGGACGCCGGAAG |
| q*Gh_A09G1589* | CGTGGAAAGAATGCAGGTG | TGAGTGGAAGCAAGCTAGTC |
| q*Gh_D01G1333* | ACACCAACTTGGTTGGGTGG | TGCCACCACCTGCAACATG |
| q*Gh_A09G0477* | CTCAGAACCAGATGCCTTGG | GTGGAAGCAAGCTAGCCCTC |
| q*Gh_A03G1024* | CTCAAGTGCCGACTTCTCC | CACTGAGGCTTTCCGCTTCC |
| q*Gh_D07G0665* | ACTCAGAGTCAGATGCCATG | GCTACTCCTCCAATTCCAAC |
| q*Gh_D09G1679* | CCAGTCCTTGCAGAATCAAG | TCATCTTTTGCCCCCTGTGC |
| q*Gh_A07G1623* | AGGCAATGTCCAGGAAAGG | TCTGCCCCAACTTCTTCCC |
| q*Gh_A10G2189* | AGATGCCATGCAGGCTCAG | TGTCCTGTGCATCGGTTTCC |
| q*Gh_A12G0596* | CAGAGGAGGCATTGTGTTGG | TGCATGAGGCACAAGTTGGG |
| q*Gh_D09G0971* | TTGCCTGGAGTAGCGCTGAC | GCACCTCACCTTAGCACACC |
| q*Gh_D05G1773* | ACAACGCCAACGTCACCTC | GGCCATGACCACCAAAAAGC |
| q*Gh_D11G2595* | GCTGCTTGTGGGAGGTGC | AGGTGGCCTTGAGCATGAC |
| q*Gh_A11G1607* | GCTGCCGAATCAACCGGTG | CTGCTCGAATCTGCCGTGG |
| q*Gh_D01G1620* | ACGGATACCCTGGTTCGGG | TCTCCCATTGCCAAACCAGC |
| q*Gh_D11G0029* | CGCTGGGTGTGTGAGTGAC | CTTTGGGACAAGCTGCCTTG |
| q*Gh_D02G2239* | GGATCAGGGTCGGAATCAGG | TCCGTCCGGATCGGAGATC |
| q*Gh_A03G1805* | GGATCAGGGTCGGAATCAGG | GGTTCCATCCGGATCGGAG |
| q*Gh_A01G1376* | AAGGATGGAGCGCTGCTACC | AGAGGCTGCAGGGACAGTAG |
| q*Gh_A08G0771* | AGTGAGTGCCTGGTCCGAG | GCAAACCACTCGAGCGCAG |
| q*Gh_D10G2297* | GTAGCCGCCTGTGAGACTG | CCAAACTCACCGGTGCAAC |
| q*Gh_A12G0352* | TGACGTGTGTCACCCGTCG | TCACAAGCGGTACCAGTGC |
| q*Gh_D03G0182* | GGGTTGTCAGTTCGCAAACC | CCAAGTTTCCACCGCCATG |
| q*Gh_D13G1816* | GGACTTGACTGCTGCAAGTG | TCCACGATTCTCACCGTTGC |
| q*Gh_A12G2428* | AGCTACGCCGGGTGTGTTAG | GCCGTCGGTTTACACGACTG |

**Figure S1**


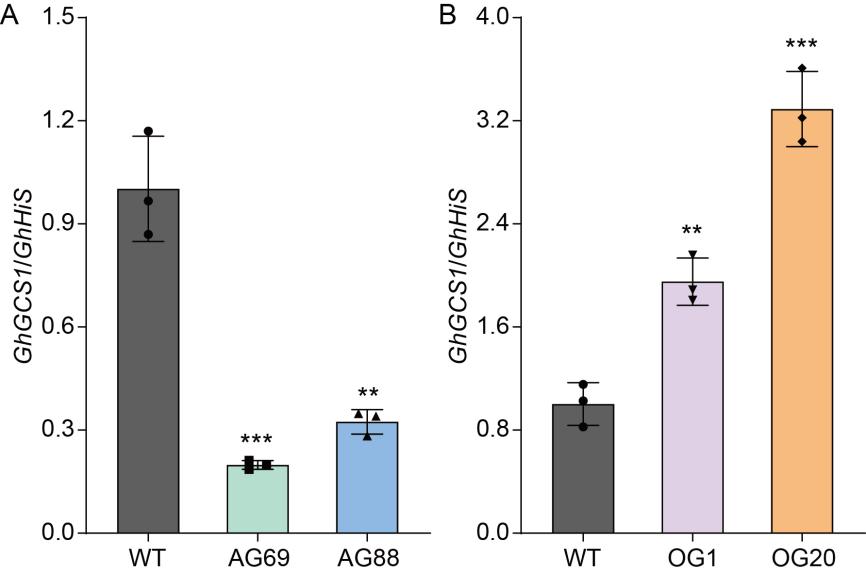


**Figure S1. The expression level of *GhGCS1* in its transgenic line root.**

(A, B)The expression level of GhGCS1 in the root of its antisense (A, AG69 and AG88) and overexpression (B, OG1 and OG20) lines detected by qRT-PCR. Error bars, ± SEM. Each analysis was repeated with three biological replicates. All *P*-values are based on two-tailed t-tests. *, *P* < 0.05; **, *P* < 0.01; ***, *P* < 0.001.

**Figure S2**

**
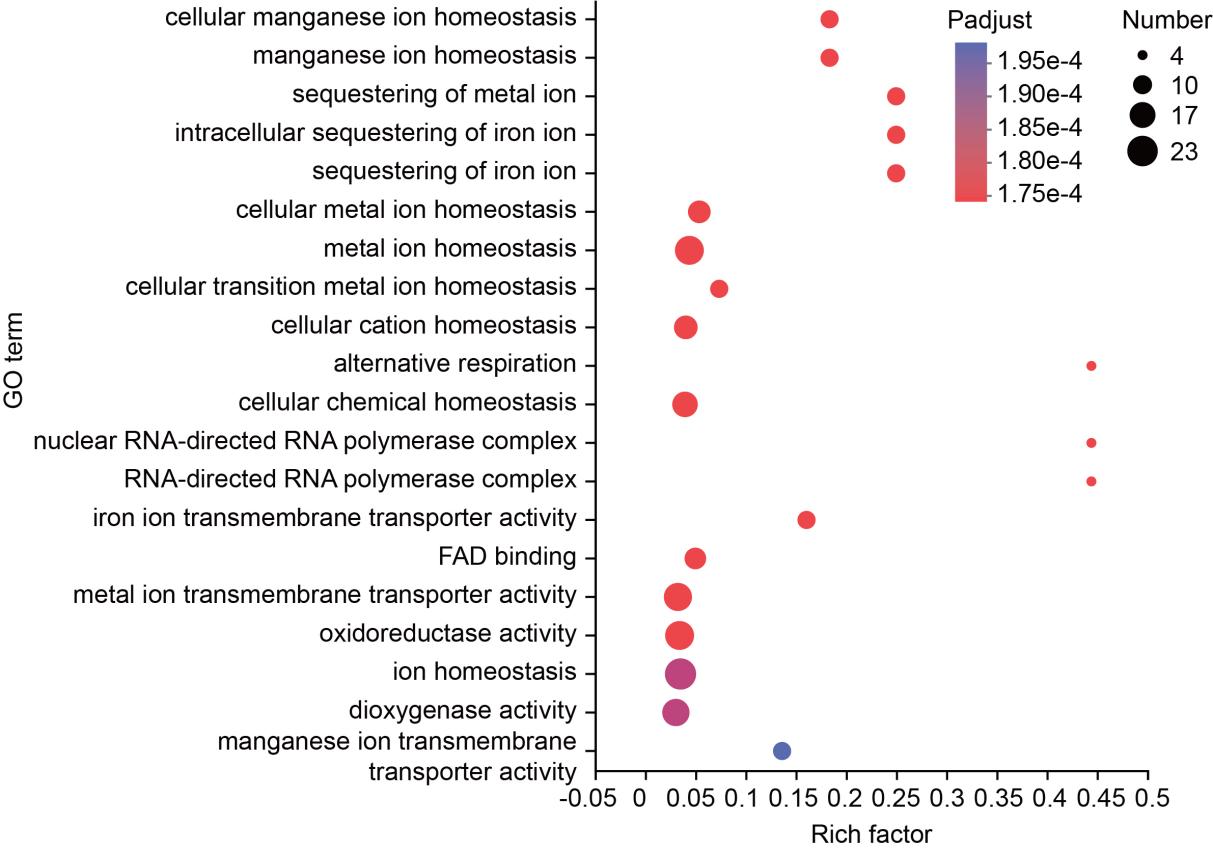
**

**Figure S2. GO enrichment analysis of DEGs.**

The scatter plot shows detailed descriptions of the pathway within the top 20 GO enrichment analysis of the 589 DEGs in *GhGCS1*-antisene roots. GO, gene ontology. Analyses of the GO enrichment were based on the condition (log2FC > 1, FDR < 0.05). The vertical axis represents the GO term, and the horizontal axis represents the ratio of the Rich factor (Sample number of genes enriched in the GO term) to the annotated gene number. The larger the Rich factor, the greater the degree of enrichment, the size of the dots indicates the number of genes in this GO term, and the color of the dots corresponds to different *P*adjust ranges.

**Figure S3**


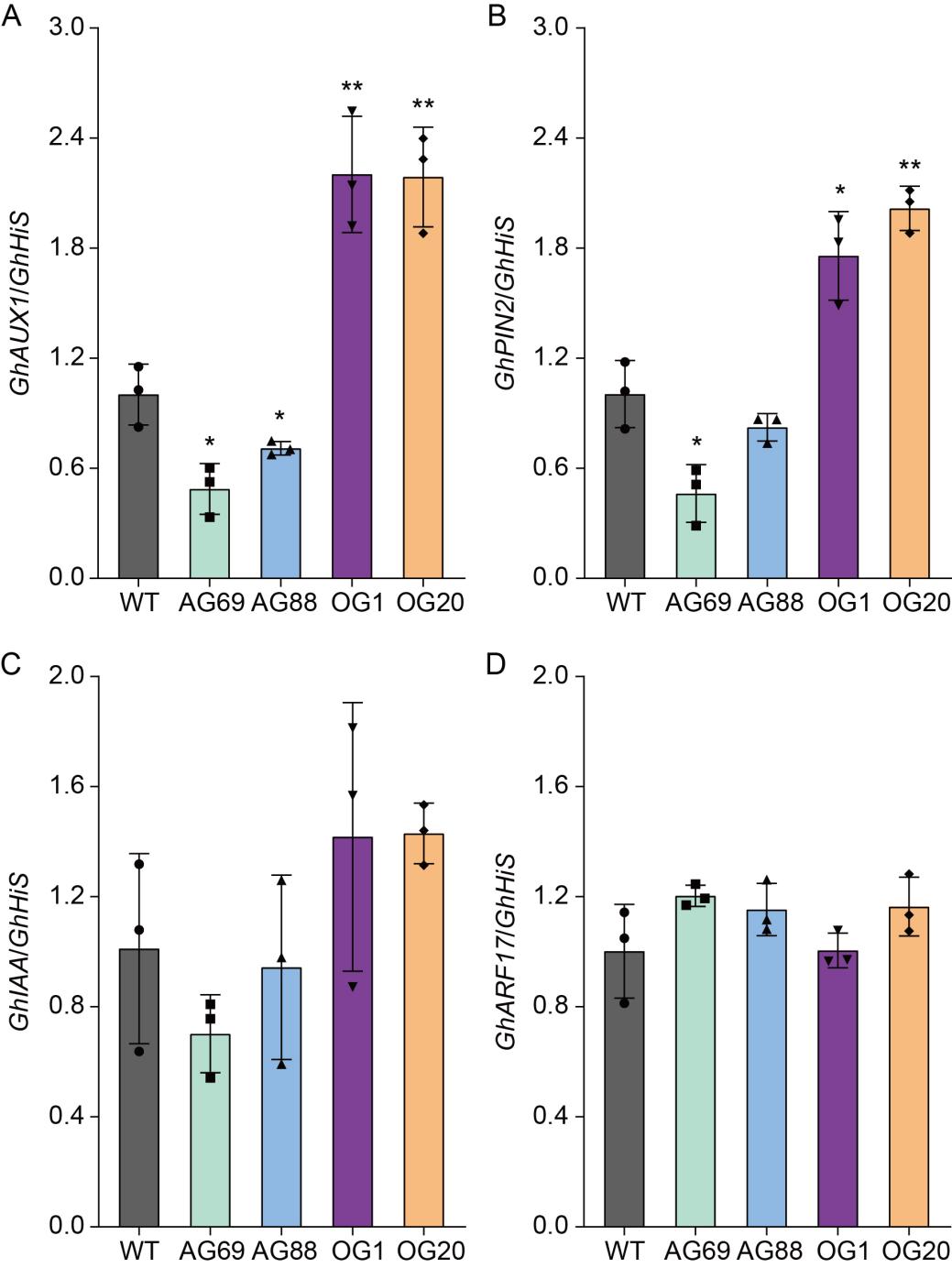


**Figure S3. The expression levels of auxin-related genes in *GhGCS1* transgenic roots.**

(A-D) The expression of *GhAUX1* (A), *GhPIN2* (B), *GhIAA* (C), and *GhARF7* (D) in *GhGCS1* transgenic roots detected by qRT-PCR. Error bars, ± SEM. Each analysis was repeated with three biological replicates. All *P*-values are based on two-tailed t-tests. *, *P* < 0.05; **, *P* < 0.01.

**Figure S4**


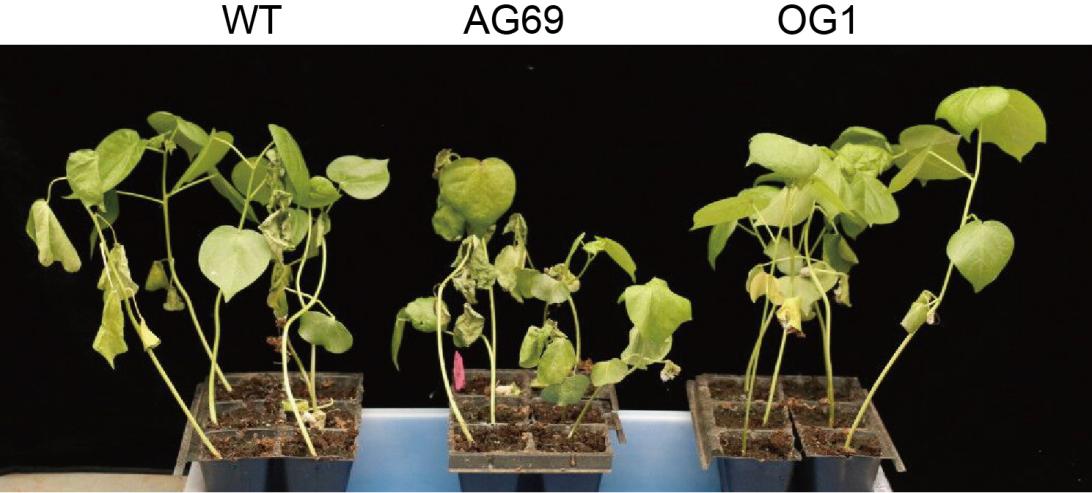


**Figure S4. *Verticillium wilt* phenotype of *GhGCS1* transgenic lines after V991 treatment.**

Phenotypes of *GhGCS1* transgenic lines after 7-day *V. dahliae* treatments. AG69, *GhGCS1-*antisense line; OG1, *GhGCS1*-overexpression line.
